# The CARE Plus study – a whole-system intervention to improve quality of life of primary care patients with multimorbidity in areas of high socioeconomic deprivation: exploratory cluster randomised controlled trial and cost-utility analysis

**DOI:** 10.1186/s12916-016-0634-2

**Published:** 2016-06-22

**Authors:** Stewart W. Mercer, Bridie Fitzpatrick, Bruce Guthrie, Elisabeth Fenwick, Eleanor Grieve, Kenny Lawson, Nicki Boyer, Alex McConnachie, Suzanne M. Lloyd, Rosaleen O’Brien, Graham C. M. Watt, Sally Wyke

**Affiliations:** Institute of Health and Wellbeing, General Practice and Primary Care, University of Glasgow, 1 Horselethill Road, Glasgow, G12 9LX UK; Population Health Sciences Division, University of Dundee, Mackenzie Building, Kirsty Semple Way, Dundee, DD2 4BF UK; Health Economics and Health Technology Assessment, Institute of Health & Wellbeing, University of Glasgow, 1 Lilybank Gardens, Glasgow, G12 8RZ UK; Robertson Centre for Biostatistics, Institute of Health and Wellbeing, University of Glasgow, Glasgow, G12 8QQ UK; Institute of Applied Health, Glasgow Caledonian University, 4th Floor George Moore Building, Cowcaddens Road, Glasgow, Lanarkshire G4 0BA UK; Institute of Health and Wellbeing, University of Glasgow, 27 Bute Gardens, Glasgow, G12 8RS UK

**Keywords:** Multimorbidity, Primary care, Deprivation, Socioeconomic, General practice, Longer consultations, Care plan, Mindfulness, Empathy, Complex intervention

## Abstract

**Background:**

Multimorbidity is common in deprived communities and reduces quality of life. Our aim was to evaluate a whole-system primary care-based complex intervention, called CARE Plus, to improve quality of life in multimorbid patients living in areas of very high deprivation.

**Methods:**

We used a phase 2 exploratory cluster randomised controlled trial with eight general practices in Glasgow in very deprived areas that involved multimorbid patients aged 30–65 years. The intervention comprised structured longer consultations, relationship continuity, practitioner support, and self-management support. Control practices continued treatment as usual. Primary outcomes were quality of life (EQ-5D-5L utility scores) and well-being (W-BQ12; 3 domains). Cost-effectiveness from a health service perspective, engagement, and retention were assessed. Recruitment and baseline measurements occurred prior to randomisation. Blinding post-randomisation was not possible but outcome measurement and analysis were masked. Analyses were by intention to treat.

**Results:**

Of 76 eligible practices contacted, 12 accepted, and eight were selected, randomised and participated for the duration of the trial. Of 225 eligible patients, 152 (68 %) participated and 67/76 (88 %) in each arm completed the 12-month assessment. Two patients died in the control group. CARE Plus significantly improved one domain of well-being (negative well-being), with an effect size of 0.33 (95 % confidence interval [CI] 0.11–0.55) at 12 months (*p* = 0.0036). Positive well-being, energy, and general well-being (the combined score of the three components) were not significantly influenced by the intervention at 12 months. EQ-5D-5L area under the curve over the 12 months was higher in the CARE Plus group (*p* = 0.002). The incremental cost in the CARE Plus group was £929 (95 % CI: £86–£1788) per participant with a gain in quality-adjusted life years of 0.076 (95 % CI: 0.028–0.124) over the 12 months of the trial, resulting in a cost-effectiveness ratio of £12,224 per quality-adjusted life year gained. Modelling suggested that cost-effectiveness would continue.

**Conclusions:**

It is feasible to conduct a high-quality cluster randomised control trial of a complex intervention with multimorbid patients in primary care in areas of very high deprivation. Enhancing primary care through a whole-system approach may be a cost-effective way to protect quality of life for multimorbid patients in deprived areas.

**Trial registration:**

Trial registration: ISRCTN 34092919, assigned 14/1/2013.

**Electronic supplementary material:**

The online version of this article (doi:10.1186/s12916-016-0634-2) contains supplementary material, which is available to authorized users.

## Background

Multimorbidity is more common, occurs earlier, and has a higher burden in patients living in high deprivation areas [1–3]. Primary care has a key role in managing multimorbidity, but the continuing existence of the ‘inverse care law’ thwarts its impact in deprived areas, thus contributing to pervasive and widening health inequalities [4–7]. Consultations are more complex in deprived areas but tend to be shorter [8–10], general practitioners (GPs) feel more stressed, and patients with complex needs are less enabled compared to those in more affluent areas [9]. Due to the mismatch between patient need and primary care capacity, GPs working in deprived areas suffer more burn-out [11].

The evidence base for enhancing the care of patients with multimorbidity is limited. A recent systematic review found only 18 randomised controlled trials (RCTs) worldwide on the management of multimorbidity in the primary care setting [12]. There is a particular dearth of evidence on multimorbidity in the context of deprivation. We have previously shown that longer consultations for deprived patients with complex needs improved enablement and reduced GP stress in a single practice in Scotland [13]. Patient-centred empathic approaches can also enhance outcomes [14, 15]. Mechanisms of action may be direct or indirect, operating, for example, through enablement and self-management support [16, 17].

Improving well-being and quality of life in patients with multimorbidity in deprived areas is likely to require multiple inputs at system, practitioner and patient level [18]. Such ‘whole-system’ approaches are likely to be even more relevant in the context of deprivation and the inverse care law. In a 4-year programme of research funded by the Scottish Government, we have developed and optimised a primary care-based whole-system intervention (CARE Plus) to improve well-being and quality of life in multimorbid patients living in high deprivation areas, based on the Medical Research Councils’ (MRC) complex intervention development framework [19] and employing quantitative [2] and qualitative [20, 21] methods to identify needs and to develop and optimise the intervention in pilot work [22].

The aim of the current study was to carry out a phase 2 exploratory cluster RCT in general practice to test the feasibility and likely cost-effectiveness of the intervention in preparation for a large-scale definitive RCT. The key research questions were:

- Is the sampling and recruitment of practices, practitioners and patients feasible?
- Is it feasible to retain patients in the trial and follow them up at 6 and 12 months in sufficient numbers (response rate of 70 % or better)?
- What is the likely benefit and cost-effectiveness of the complex intervention when trialled in a large, definitive, cluster randomised trial?

## Methods

### Study design and participants

We performed a phase 2 cluster RCT to evaluate the likely effectiveness and cost-effectiveness of the CARE Plus intervention on patient outcomes and to assess engagement and retention of practices and patients. Participating general practices formed the cluster. Our aim was to recruit eight practices. Practices were eligible if they were in Glasgow and in the 100 practices in Scotland (which has approximately 1000 practices in total) serving the most deprived patients, based on the percentage of registered patients in the 15 % most deprived postcodes. Practices were excluded if they were unwilling to deliver the intervention or could not attend the first training session. The 76 eligible practices were sent details of the study; 26 replied within 2 weeks, 12 were willing to participate, and eight were selected based on previous participation in quality-improvement initiatives.

Patients were eligible if they were aged between 30 and 65 years and had two or more long-term conditions. The type of condition was not specified. Before randomisation, participating practices were asked to identify approximately 25 patients per practice who met the above inclusion criteria and who they believed would benefit from and participate in the intervention. Exclusion criteria were (a) unable to give informed consent including those with severe learning disability, severe active mental health problems (active psychosis, schizophrenia, bipolar illness, psychotic depression, severe depression including active suicidal ideation), severe dementia, or other severe cognitive impairments; (b) terminally ill or considered by their GP as likely to die within next 12 months; and (c) unable to understand spoken and written English.

Patients were recruited and baseline data collected face-to-face before randomisation after written informed consent was obtained. Patients could choose whether to complete the follow-up questionnaires by post, telephone or face-to-face. At follow-up, patients who did not return mailed questionnaires within 10 days were telephoned to establish their continuing participation and ascertain again their preferred method for completing the questionnaire. On completing each questionnaire, patients were given a £5 gift voucher as a token of appreciation. Details of the measures used are shown in Additional file 1. Implementation of CARE Plus consultations (intervention fidelity) was estimated from the details recorded on the CARE Plus care plan (see below), and the patient-reported questionnaire data (post-consultation and follow-up).

### Randomisation and masking

The unit of randomisation was practice; the randomisation schedule was generated by an independent statistician within the Robertson Centre for Biostatistics after patients gave consent and baseline data were collected. Practices were ordered according to list size and randomised to intervention or control within consecutive pairs, to avoid an imbalance in list size between randomised groups. Due to the intervention design it was not possible to mask intervention allocation from the practitioners or the patients once the trial had started. The research nurses who collected outcome data were blind to the treatment allocation at all stages, as were the trial analysts.

### Development and optimisation of the CARE Plus intervention

The intervention was developed according to MRC complex intervention guidelines [19]. This included qualitative interviews with GPs and practice nurses working in, and multimorbid patients living in, deprived areas of Glasgow [20,210] to understand how primary care might better respond. From these, and the available evidence, we drew up an outline of a whole-system intervention which included longer consultations, a structured patient-centred empathic approach, relational continuity, practitioner training and support, and patient self-management support. We then gathered views on the proposed intervention from multimorbid patients, GPs and practice nurses in deprived areas and from patient advocacy groups [22]. The intervention was then piloted in two eligible practices not in the main trial and further optimised [22].

The final CARE Plus intervention involved:

1. Changes to practice systems to allow longer consultations (30–45 minutes) and relational continuity with eligible multimorbid patients. Each practice decided what changes would be necessary to allow this; provided that they achieved the intended aims, practices were allowed to decide on how to implement this in their particular organisational context.
2. Group-based practitioner support and training to use the longer CARE Plus structured consultations to carry out a holistic assessment, including identification of patient concerns and priorities, a focus on self-management, and agreeing on a care plan (the CARE Approach [23]).
3. Additional patient self-management support materials (mindfulness-based stress management CDs, a cognitive behavioural therapy-derived self-help booklet) and written material (also supplied on a CD) about the intervention and the self-help material (available on request from the corresponding author).

Practitioners were encouraged to link patients with relevant local resources and community services when appropriate. Follow-up consultations were arranged as required with the same practitioner. Practitioners were asked to give participating patients the self-management support pack. Practitioners documented the details of the consultations in the care plan, including consultation length, problems explored and patient-identified goals.

Practitioner training and support for those in the CARE Plus group involved three half-day meetings; one at the start of the intervention and the other two spaced out over the remaining 12 months. The intervention was explained and discussed at the first meeting. Participants set shared goals, and made personal learning plans based on gaps in knowledge and/or skills. At the second and third meetings, the group set shared goals for the session and reviewed progress in the intervention. Case-based discussions were encouraged to facilitate shared learning. Two experienced practitioners facilitated the meetings, one an academic GP (SWM) and the other a psychiatrist with extensive expertise in managing patients with complex needs. The sessions included 20–30 minutes of mindfulness-based stress reduction for practitioners [24].

### Outcomes

The primary patient-reported outcome measures were health-related quality of life (EQ-5D-5L) [25] and well-being (W-BQ12) [26]. The EQ-5D-5L was calculated as a single preference-weighted utility score. The W-BQ12 has three components measured in its 12 items: negative well-being, positive well-being, and energy. These component scores can be combined to give an overall general well-being score. Secondary patient-reported outcomes were anxiety and depression (Hospital Anxiety and Depression Scale, HADS [27]), self-efficacy [28] and self-esteem [29]. The baseline patient questionnaire included demographic characteristics. Deprivation was estimated by the Scottish Index of Multiple Deprivation (SIMD) [30]. The level (number of conditions) and burden (effect on daily life) of multimorbidity was assessed at baseline [31] and expressed as the average mean score. The level of engagement and retention of practices and patients in the trial was also measured in terms of recruitment and retention rates. Qualitative interviews with practitioners and patients were also conducted but will be reported in a separate paper.

Health service utilisation and prescribing data were extracted from the electronic medical records of participating patients for the 12 months before and 12 months after the study began. This included all consultations within the practice (with any healthcare professional, whether face-to-face or by phone), and all out-patient consultations and in-patient admissions.

Practitioners’ views on the training and support sessions were collected 6 weeks after the last session using a scale from 0 (not beneficial) to 4 (extremely beneficial) in terms of overall benefit of training in meeting collective goals, meeting personal goals, helping with participation in trial, peer support, and helping deal with challenges.

### Statistical and economic analysis

Patient characteristics at baseline were summarised by intervention group as mean (standard deviation [SD]) or frequency (per cent) for continuous and categorical outcomes, respectively. Differences between the groups at baseline were examined using regression models that adjusted for clustering at the practice level (linear models for continuous variables and logistic models of dichotomous variables); variables with significant differences at baseline (at the 10 % significance level) were adjusted for in the analysis of outcome measures. Change from baseline in the outcome measures was calculated as value at follow-up (6 months or 12 months) minus baseline value. Differences between the intervention and control groups in the change from baseline values was tested separately at each follow-up point using linear regression methods that adjusted for the clustering of patients within practices as a random effect. In addition, these models adjusted for baseline value, age, sex and those baseline characteristics found to be significantly different between the groups. From these models, the mean difference (95 % confidence interval [CI]) and *p* value are presented; effect sizes (95 % CI) have also been derived for each outcome measure by dividing the mean difference by the SD of the baseline measure for all participants.

In addition, for the EQ-5D data, the area under the curve (AUC) was derived at 6 and 12 months using the available data. The AUC was analysed in a linear model as described above. A within-trial cost-utility analysis was carried out based on the EQ-5D-5L utility scores, and on health service utilisation in control and intervention groups. The evaluation was undertaken from the NHS and Personal Social Service perspective favoured by the National Institute for Health and Care Excellence (NICE) [32]. The analysis estimated the cost-effectiveness of the CARE Plus intervention compared with usual care over the period of the trial (12 months). Differences in the average utility change between the intervention and comparison groups gave an estimate of the quality-adjusted life years (QALYs) gained from the intervention. The longer-term cost-effectiveness of CARE Plus, using a discount rate of 3.5 % [32], was estimated by extrapolating the within-trial results over 2 years, and included a probabilistic sensitivity analysis and value of information analysis to account for uncertainty. Full details of the analysis and further references can be found in Additional file 2.

### Sample size calculation

As the current study was a planned phase 2 exploratory trial intended to evaluate feasibility and to estimate the required power for a phase 3 trial, we did not base the sample size on a power calculation, given the paucity of evidence on which to do this.

### Data access

The trial statisticians (AM and SL) had full access to the data. EF, EG, KL and NB had access to the economic data. All other authors contributed to data interpretation.

### Trial registration and ethical approval

The trial was registered with Current Controlled Trials (ISRCTN 34092919). Due to an administrative error, the registration was not applied for until the 28 November 2012 (6 weeks after the trial started) and was attained on the 14 January 2013. Ethical approval was granted by the West of Scotland Research Ethics Service, reference number 11/WS/0031, prior to the start of the trial.

## Results

### Baseline characteristics of practices and patients

The practices in the intervention and control groups were of similar size in terms of the mean number of registered patients, with 3605 (SD 1338.1) patients in the intervention versus 3710 (SD 1748.9) in the control. Participating practices ranged from the fifth to the 62nd most deprived practices in Scotland. Mean deprivation (SIMD) scores for all registered patients were 52.35 (SD 5.70) in the intervention practices, compared to 49.23 (SD 8.93) in the control practices. Deprivation scores for patients in the trial were not significantly different from the practice means (Table 1). Participating patients had a mean age of 52 years with a mean of five long-term conditions each. Patients’ characteristics and baseline measures did not differ significantly between the intervention and control groups (see Table 1 and Additional file 1).

**Table 1:** Participating patient characteristics

Characteristics	Usual care (*N* = 76)	CARE Plus (*N* = 76)	*p* value for difference between groups
Sex:			
Female	39 (51 %)	46 (61 %)	0.35
Male	37 (49 %)	30 (39 %)	
Age:			
Mean (SD)	53.1 (8.0)	51.9 (9.6)	0.52
<50	22 (29 %)	28 (37 %)	
50–59	34 (45 %)	28 (37 %)	
≥ 60	20 (26 %)	20 (26 %)	
Index of Multiple Deprivation:			
Mean (SD)	52.8 (21.9)	49.8 (22.0)	0.54
Q1 (least deprived)	2 (3 %)	2 (3 %)	
Q2	1 (1 %)	3 (4 %)	
Q3	4 (5 %)	5 (6 %)	
Q4	7 (9 %)	8 (11 %)	
Q5 (most deprived)	61 (82 %)	57 (76 %)	
Number of chronic conditions:			
Mean (SD)	5.1 (2.1)	4.8 (2.6)	0.51
2	7 (9 %)	14 (18 %)	
3	12 (16 %)	11 (14 %)	
4	14 (18 %)	18 (24 %)	
5	13 (17 %)	12 (16 %)	
≥6	30 (40 %)	21 (28 %)	
Burden of multimorbidity			
Mean (SD)	3.1 (1.0)	3.2 (1.1)	0.40
Primary patient outcomes			
EQ5D-5 L	0.419 (0.325)	0.419 (0.318)	0.99
W-BQ12 General Well-being Score (total)	16.0 (8.0)	14.3 (9.0)	0.29
W-BQ12 Negative Well-being Score	5.1 (3.5)	6.3 (4.1)	0.14
W-BQ12 Energy Score	3.2 (2.7)	3.1 (2.8)	0.86
W-BQ12 Positive Well-being Score	5.8 (3.5)	5.7 (3.7)	0.79

Summary statistics are presented as mean (SD) or number (per cent)

### Primary patient-reported outcomes

**Fig. 1:**
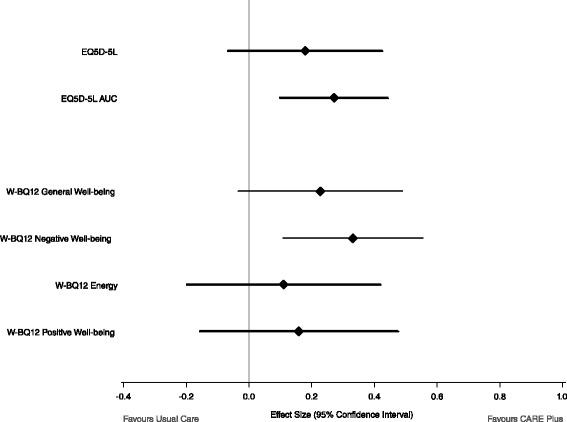
Primary outcomes: quality of life and well-being at 12 months

As shown in Table 2, after adjusting for baseline variables there was a significant difference in EQ-5D-5L utility score at 6 months (*p* = 0.039) in favour of the CARE Plus group, although this was no longer significant at 12 months (*p* = 0.15). There was a significant difference in one of the three components of the W-BQ12 (*p* = 0.004) in favour of the CARE Plus group at 12 months, signifying a reduction in negative well-being relative to controls. Positive well-being, energy and general well-being (the combined score of the three components) were not significantly influenced by the intervention at 6 or 12 months, though the trend was in favour of the CARE Plus group by 12 months (Fig. 1).

**Table 2:** Primary outcomes at 6 and 12 months in control and intervention groups

Outcomes	Change from baseline		Adjusted^a^ mean difference (95 % CI)	Effect size (95 % CI)	*p* value
	Usual care	CARE Plus			
Primary outcomes:					
6 months					
EQ5D-5 L	−0.08 (0.27)	0.04 (0.25)	0.13 (0.01, 0.25)	0.38 (0.00, 0.75)	0.039
W-BQ12 General Well-being	−1.3 (5.1)	−0.2 (6.5)	0.70 (−2.45, 3.85)	0.08 (−0.28, 0.44)	0.66
W-BQ12 Negative Well-being	1.0 (2.8)	0.0 (2.9)	−0.69 (−1.63, 0.24)	0.18 (−0.06, 0.42)	0.14
W-BQ12 Energy	0.7 (2.8)	0.2 (2.7)	−0.42 (−1.85, 1.01)	−0.15 (−0.65, 0.36)	0.56
W-BQ12 Positive Well-being	−0.9 (2.8)	−0.5 (3.5)	0.66 (−0.31, 1.64)	0.18 (−0.09, 0.46)	0.18
12 months					
EQ5D-5 L	−0.02 (0.28)	0.02 (0.26)	0.06 (−0.02, 0.14)	0.14 (−0.10, 0.39)	0.15
W-BQ12 General Well-being	−1.6 (5.6)	0.4 (6.8)	1.99 (−0.27, 4.24)	0.23 (−0.03, 0.49)	0.083
W-BQ12 Negative Well-being	1.2 (2.8)	−0.2 (2.5)	−1.30 (−2.16, −0.43)	0.33 (0.11, 0.55)	0.0036
W-BQ12 Energy	0.6 (2.4)	0.6 (2.9)	0.31 (−0.55, 1.17)	0.11 (−0.20, 0.42)	0.47
W-BQ12 Positive Well-being	−0.8 (3.3)	−0.6 (3.8)	0.57 (−0.56, 1.70)	0.16 (−0.16, 0.48)	0.32

^a^Mean differences were adjusted for baseline score, age, gender and Hospital Anxiety and Depression Scale caseness - positive effects sizes indicate a difference in favour of the intervention group.

*CI* confidence interval

### Cost-utility analysis based on EQ-5D-5L

**Fig. 2:**
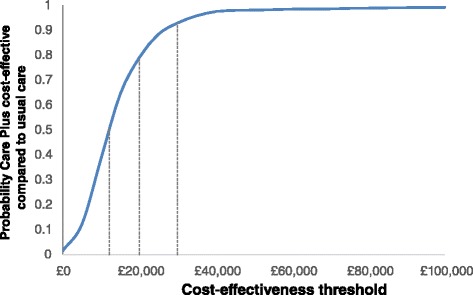
Economic cost-effectiveness acceptability curve for CARE Plus intervention

When measured as AUC, there was a significant difference between the EQ-5D-5L scores over the 12-month period, in favour of the CARE Plus group (*p* = 0.002; effect size 0.36) (Fig. 1). The within-trial cost-utility analysis estimated the immediate impacts (in terms of costs and effects) associated with the CARE Plus intervention compared with usual care. The total costs associated with the CARE Plus group were estimated to be £312,449 compared to total costs for usual care of £243,793. The CARE Plus group was associated with an increase in total costs of £82,989 compared to a slight increase in costs for usual care of £487, giving an incremental cost of £82,501 and an adjusted mean difference of £929 (95 % CI £86–£1788) per participant. CARE Plus was more effective in terms of QALYs with a gain of 0.076 QALYs (95 % CI 0.028–0.124) over the 12 months of the trial. As a result, the CARE Plus intervention was associated with an incremental cost-effectiveness of £12,224 per QALY gained. The cost-effectiveness acceptability curve (Fig. 2) illustrates the probability that the CARE Plus intervention was cost-effective for any given value of the cost-effectiveness threshold. For a cost-effectiveness threshold of £20,000/QALY, the probability that CARE Plus was cost-effective (compared to usual care) was 0.79, and this rose to 0.93 for a cost-effectiveness threshold of £30,000/QALY. Modelling of estimated effects 2 years beyond the trial period suggested that this cost-effectiveness would be likely to continue in the longer term (see Additional file 2).

### Secondary patient outcomes

None of the secondary outcomes were significantly different between intervention and control groups, though anxiety (*p* = 0.08) and depression (*p* = 0.06) measured by the HADS were of borderline significance in favour of the CARE Plus group, although the effects sizes were relatively small. Self-esteem and self-efficacy showed the least evidence of change. Full details of all outcomes are shown in Additional file 1.

### Engagement and retention

**Fig. 3:**
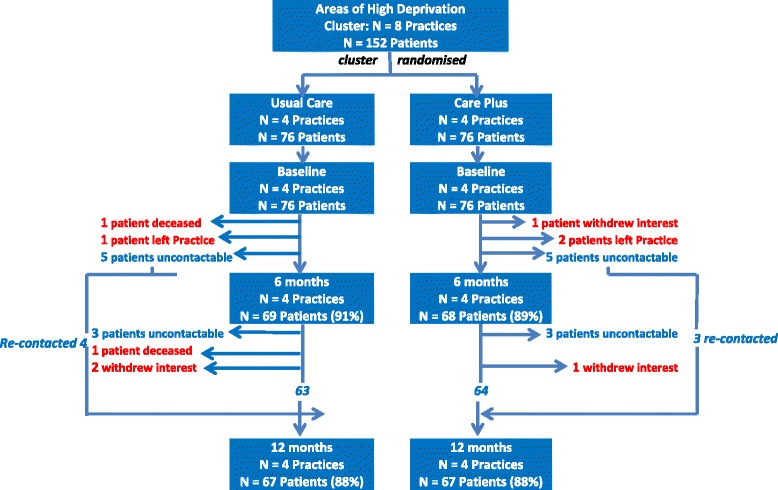
Trial flow chart

All eight practices remained in the study for the 12-month study period. Outcome data were collected on 90 % of patients at 6 months (91 % in the intervention group and 89 % in the control group) and 88 % (in both groups) at 12 months (Fig. 3). Practices in the intervention group engaged well with the training and support meetings, with all nine GPs and practice nurses involved in delivering the intervention attending training sessions 1 and 2, and 78 % (7/9) attending the final training session. Seventy eight per cent (7/9) of participating staff responded to the evaluation of the training, reporting mean scores (out of 4) of 3.4 (SD 0.54) for overall benefit of training, 3.6 (SD 0.54) for meeting collective goals, 3.3 (SD 0.95) for meeting personal goals, 3.4 (SD 0.79) for helping with participation in the trial, 3.4 (SD 0.79) for peer support, and 3.3 (SD 0.95) for helping deal with challenges.

Implementation of CARE Plus consultations (intervention fidelity) was estimated from the care plan and the patient report data. Both sources of data confirmed that the CARE Plus patients received substantially longer initial consultations; with a mean length of 36.9 minutes (SD 9.8), according to the care plan, and a mean 34.1 minutes (12.7) according to the patient report. The mean consultation length for the second and third consultations were 29.4 (SD 13.0) and 22.3 (SD 8.4), respectively. The total amount of time in the CARE Plus consultations per patient was on average 69.2 (SD 30.18, *n* = 54). The number of CARE Plus consultations recorded per patient varied from one to six; 22 % received one consultation, 40 % received two, and 38 % receiving three or more. Patients saw the same practitioner in all CARE Plus consultations. The mean number of consultations over the 12-month period was eight in the CARE Plus group (two CARE Plus and six non-CARE Plus consultations) and eight in the usual care group. Healthcare practitioners reported giving the self-management pack to 97 % of patients over the course of the CARE Plus intervention; 71 % of patients reported having received this in the 12-month follow-up questionnaire. Practitioners reported signposting patients to local community self-management resources in 49 % of CARE Plus consultations; 45 % of patients reported receiving this advice.

Two patients died in the usual care group during the course of the study; one from the sequelae of a stroke after several months in hospital, and one (who was under the care of a cardiologist and a diabetologist) from an acute myocardial infarction.

## Discussion

A primary care-based complex intervention to improve well-being and quality of life targeted at very deprived patients with multimorbidity was tested in a phase 2 exploratory cluster RCT. The intervention consisted of system changes to allow structured longer consultations and relationship continuity, group-based training and support for practitioners, and self-management support material for patients. The findings provide preliminary evidence of effectiveness and cost-effectiveness of the intervention and demonstrate the feasibility of carrying out a high-quality trial in a very challenging context. The main effect of the intervention appeared to be to limit the decline in quality of life and well-being seen in the control group.

A recent pilot study of a culturally sensitive well-being intervention for underserved patients in primary care showed some evidence of acceptability and efficacy but had major problems with recruitment and engagement [33]. A key component of our intervention was substantially longer consultations, adding to the limited evidence base on the possible benefits of more time for such patients [34, 35]. A large primary care-based RCT that aimed to enhance self-management in general practice in a relatively high deprivation setting with multimorbid patients failed to show any benefit [36]. Unlike the CARE Plus study, it did not include longer consultation time with the GPs. Thus, increasing consultation length may be crucial in delivering better care, including self-management support, in this population. The effect sizes of the primary outcomes that were significant in the current study (0.38 for EQ-5D-5L at 6 months, 0.33 for W-BQ12 at 12 months) were slightly larger than recently reported for a collaborative care approach for multimorbid patients in deprived areas in England (adjusted effect size 0.30) [37]. A recent small feasibility RCT (*n* = 50) in Ireland of an occupational therapist-led self-management support intervention for multimorbid patients also reported benefit [38]. However, neither study reported cost-effectiveness. The CARE Plus intervention was associated with an incremental cost-effectiveness of £12,224 per QALY gained, well below the threshold range of £20,000–£30,000 per QALY used by NICE to recommend NHS implementation in England and Wales [39]. Modelling suggested that this cost-effectiveness would be likely to continue in the longer term (see Additional file 2).

The importance of developing and optimising the intervention along MRC guidelines using mixed methods and working in partnership with all the key stakeholders should be emphasised [19]. Our intervention was developed systematically in a programme of work that (a) used large-scale epidemiological data to identify the target population [2]; (b) used qualitative methods to gain the views and perspectives of practitioners and patients to identify the main problems and potential solutions [20, 21]; and (c) consulted a range of stakeholders on the likely acceptability of the initial intervention, and used these views and pilot studies to further optimise the intervention [22]. Although this process took a considerable amount of time (54 months including the exploratory RCT), we believe this was very important in terms of ‘co-designing’ and refining a bespoke complex intervention that was acceptable to practitioners and patients.

A major strength of this study is the high level of retention that we achieved in terms of practices and patients who are conventionally regarded as difficult to engage in research. The amount of work required to collect the patient outcome data was substantial (see Additional file 1: Table S4) Given the small number of studies conducted in primary care on multimorbid patients in general, and in areas of high deprivation in particular, this study is important in demonstrating that such research can be done to a high level of quality in such settings if adequately resourced. Given the context of an exploratory phase 2 trial intended to examine feasibility and provide an initial estimate of effectiveness to power a definitive trial, inevitable weaknesses include the relatively small number of practices and patients involved and relatively short trial period.

A possible weakness in the trial was the possibility of un-blinding of the research nurses who did face-to-face or telephone follow-up at 6 months and 12 months. This was not measured, but almost two-thirds of follow-up was done by postal questionnaire (see Additional file 1: Table S4) in which un-blinding was not possible.

Our economic analysis took a standard approach and did not attempt to measure any benefits of the intervention in terms of family and community benefit resulting from the improved quality of life and well-being in these vulnerable patients. There may have also been benefits for practitioners in being supported to deliver more effective care, which has the potential to enhance practitioner well-being and reduce practitioner stress and burn-out [9, 11], and perhaps even enhance workforce retention in deprived areas.

Proceeding to a definitive phase 3 trial would appear to be feasible and warranted, given the high burden of multimorbidity in deprived areas on healthcare costs [39] and the very limited evidence base on interventions [12, 40, 41]. Our value of information analysis also suggests this would be good value for money in terms of research investment (see Additional file 2).

## Conclusion

A complex whole-system intervention in primary care to enhance well-being and quality of life in patients with multimorbidity in deprived areas has been developed and evaluated in a cluster randomised phase 2 trial, demonstrating feasibility and plausible benefit. As far as we are aware, this is the first study in the world of its kind. It shows that high-quality RCTs of complex interventions can be done in patients who have the highest needs due to complex problems and living in areas of extreme deprivation. Enabling practices in deprived areas to provide longer and more patient-centred care for multimorbid patients may protect quality of life in a cost-effective way.

### Abbreviations

AUC, area under the curve; CI, confidence interval; GP, general practitioner; HADS, Hospital Anxiety and Depression Scale; MRC, Medical Research Council (United Kingdom); NICE, National Institute for Health and Care Excellence (United Kingdom); QALYs, quality-adjusted life years; RCT, randomised controlled trial; SD, standard deviation; SIMD, Scottish Index of Multiple Deprivation.

## Additional files

Additional file 1: Baseline measures and additional data. (DOCX 221 kb) Additional file 2: Economic analysis. (DOCX 27 kb)
