# Supplementary material for: The CARE Plus study – a whole-system intervention to improve quality of life of primary care patients with multimorbidity in areas of high socioeconomic deprivation: exploratory cluster randomised controlled trial and cost-utility analysis
Source: BMC Med. 2016 Jun 22;14:88. doi: 10.1186/s12916-016-0634-2 (PMC4916534; doi:10.1186/s12916-016-0634-2)
Supplement: Additional file 1: — Baseline measures and additional data. (DOCX 221 kb) [file 12916_2016_634_MOESM1_ESM.docx]

**Additional file 1**

**Additional results: secondary outcomes**

**Table S.1.Patient Characteristics (Secondary outcomes)**

| Characteristic | Usual Care  (N = 76) | CARE Plus  (N = 76) | p-value for difference between groups |
| --- | --- | --- | --- |
| **Secondary Patient Outcomes** |  |  |  |
| Stanford Self-Efficacy Scale | 4.5 (2.2) | 4.9 (2.6) | 0.35 |
| Rosenberg’s Self-Esteem Scale | 16.3 (6.5) | 16.3 (6.3) | 0.94 |
| HADS Anxiety Score | 10.9 (5.1) | 11.1 (5.7) | 0.87 |
| HADS Depression Score | 8.5 (4.7) | 9.4 (5.7) | 0.37 |
| HADS Caseness | 19 (25%) | 32 (42%) | 0.074 |
|  |  |  |  |

**Table S.2. Baseline Chronic Conditions**

| Chronic Condition | Usual Care  (N = 76) | CARE Plus  (N = 76) |
| --- | --- | --- |
| Anxiety / Depression | 52 (68%) | 49 (65%) |
| High Blood Pressure | 48 (63%) | 39 (51%) |
| Back Problems | 35 (46%) | 35 (46%) |
| Arthritis | 27 (36%) | 31 (41%) |
| Asthma | 30 (40%) | 27 (36%) |
| Diabetes | 31 (41%) | 19 (25%) |
| Angina / Heart Attack | 22 (29%) | 25 (33%) |
| Migraine | 17 (22%) | 26 (34%) |
| Irritable Bowel Syndrome | 19 (25%) | 18 (24%) |
| Eczema / Psoriasis | 15 (20%) | 11 (15%) |
| Chronic Bronchitis | 10 (13%) | 12 (16%) |
| Stroke / Mini-Stroke | 10 (13%) | 11 (15%) |
| Thyroid Problem | 8 (10%) | 7 (9%) |
| Kidney Disease | 6 (8%) | 7 (9%) |
| Liver Disease | 5 (7%) | 3 (4%) |
| Cancer | 0 (0%) | 6 (8%) |
| Heart Failure | 4 (5%) | 2 (3%) |
| Other | 45 (59%) | 35 (46%) |

**Table S.3. Secondary outcomes at 6 and 12 months in control and intervention groups**

| Outcomes | | | Change from baseline | | Adjusted* mean difference (95% CI) | Effect size (95% CI) | p-value |
| --- | --- | --- | --- | --- | --- | --- | --- |
|  |  |  | Usual Care | CARE Plus |  |  |  |
| Primary Outcomes: | | |  |  |  |  |  |
|  | 6 months | |  |  |  |  |  |
|  |  | Stanford Self-Efficacy Scale | 0.06 (1.60) | 0.02 (2.54) | 0.13 (-0.66, 0.91) | 0.05 (-0.27, 0.37) | 0.75 |
|  |  | Rosenberg’s Self-Esteem Scale | -0.8 (5.3) | -0.5 (4.7) | 0.28 (-1.48, 2.03) | 0.04 (-0.24, 0.32) | 0.76 |
|  |  | HADS Anxiety | 0.6 (3.1) | -0.1 (3.9) | -0.50 (-2.07, 1.06) | 0.09 (-0.20, 0.39) | 0.52 |
|  |  | HADS Depression | 1.4 (2.6) | 0.6 (3.8) | -0.67 (-1.80, 0.47) | 0.13 (-0.09, 0.35) | 0.25 |
|  |  |  |  |  |  |  |  |
|  | 12 months | |  |  |  |  |  |
|  |  | Stanford Self-Efficacy Scale | 0.32 (1.85) | 0.05 (2.43) | 0.07 (-0.69, 0.83) | 0.03 (-0.28, 0.34) | 0.85 |
|  |  | Rosenberg’s Self-Esteem Scale | -1.0 (4.4) | -0.8 (5.4) | 0.74 (-0.96, 2.45) | 0.11 (-0.15, 0.37) | 0.39 |
|  |  | HADS Anxiety | 0.8 (2.7) | -0.1 (3.4) | -0.91 (-1.93, 0.12) | 0.17 (-0.02, 0.36) | 0.084 |
|  |  | HADS Depression | 1.9 (3.5) | 0.6 (4.0) | -1.25 (-2.53, 0.03) | 0.24 (-0.01, 0.48) | 0.056 |
|  |  | |  |  |  |  |  |

| Data Collection Period | Total Number of Patients with who Contact was Attempted | Number (%) of Patients for whom Data were Collected | Telephone Contact Attempted | | Data Collection Method | | |
| --- | --- | --- | --- | --- | --- | --- | --- |
|  |  |  | Total Number All Patients | Mean Number Per Patient | Number (%) by Face-to Face Interview | Number (%)by Telephone Interview | Number (%) by Post |
| Baseline | 226 | 152 (6) | 2025 | 8.96 | 152 (100) |  |  |
| 6 month follow-up | 147* | 137 (90) | 835 | 5.68 | 46 (33.6) | 13 (9.5) | 78 (56.9) |
| 12 month follow-up | 148 | 134 (88) | 1056 | 7.14 | 29 (21.6) | 17 (12.7) | 88 (65.7) |
|  |  |  |  |  |  |  |  |
| * for 5 patients no contact attempt was made (1 deceased,3 had left Practice, 1 recently bereaved) | | | | | | | |

**Table S.4. Workload involved in patient recruitment and retention**

**Figure S.1. Secondary Outcomes; self efficacy, self esteem, anxiety and depression**
