# Supplementary material for: The CARE Plus study – a whole-system intervention to improve quality of life of primary care patients with multimorbidity in areas of high socioeconomic deprivation: exploratory cluster randomised controlled trial and cost-utility analysis
Source: BMC Med. 2016 Jun 22;14:88. doi: 10.1186/s12916-016-0634-2 (PMC4916534; doi:10.1186/s12916-016-0634-2)
Supplement: Additional file 2: — Economic analysis. (DOCX 27 kb) [file 12916_2016_634_MOESM2_ESM.docx]

# Additional file 2

# Economic Evaluation

The aim of the economic analyses was to determine the cost-effectiveness of CARE Plus compared with usual care. Throughout the analyses, the control group was used as the source of data for the usual care group. The evaluation was undertaken from the NHS and Personal Social Service perspective favoured by the National Institute for Health and Care Excellence^1^.

**Analyses**

An initial, short-term, analysis estimates the cost-effectiveness of CARE Plus compared with usual care over the period of the trial. This analysis requires the assumption that there are no differences in costs or effects between the intervention and control groups beyond the 12 month trial follow-up period. A second, longer term, analysis employs a model to estimate additional costs and benefits over the participants’ lifetime in order to provide an estimate of the longer term cost-effectiveness of CARE Plus compared with usual care. Further details of each analysis are provided in the sections below.

**Within-trial analysis**

The aim of the within-trial analysis was to estimate the immediate impacts (in terms of costs and effects) associated with the CARE Plus intervention in order to establish the cost-effectiveness of the intervention compared with usual care. The within-trial analysis compared the CARE Plus intervention group with the control group in terms of (a) costs incurred over the 12 month period and (b) QALYs gained over the 12 month period.

***Resource Use and Costs***

The cost calculation included the resources required to provide the CARE Plus intervention and the changes in health care utilisation, positive or negative, resulting from the intervention including medication use and health care consultations (table 1).

**Table 1. Resource costs**

| **RESOURCE** | **UNIT COST** | **SOURCE** |
| --- | --- | --- |
| Cost of CARE Plus intervention | General Practitioner/hour (£221) or Practice Nurse/hour (£53) | PSSRU 2012 |
| Prescriptions | Cost per prescribed item | BNF 2012 |
| GP consultation surgery | £43 per consultation (average 11.7 mins) | PSSRU 2012 |
| GP telephone call | £26 per call (average 7.1 mins) | PSSRU 2012 |
| GP home visit | £110 per home visit (average 23.4 mins) | PSSRU |
| Primary care nurse | £53 per hour face-to-face  £41 per hour (telephone etc)  (average 15.5 mins) | PSSRU 2012 |
| Out of hours GP | £282 per hour | PSSRU 2012 |
| A&E | £104 (average cost per attendance) | ISD Scotland 2012 |
| Outpatients | Average cost per attendance as defined by ward and where possible hospital | ISD RO4X 2012 and NHS reference costs |
| Inpatient visits | Cost per case defined by ward and where possible hospital | ISD RO4X 2012 and NHS reference costs |
| Lab/radiology costs | Net cost per examination/cost per specimen | ISD R130X 2012 |

**Resource use and costs**

Health care utilisation was collected, through an audit of patient records, for the 12 month period prior to baseline and the 12 months post randomisation. The period prior to baseline was used to ensure that there were no differences between the intervention and control groups, in terms of resource use, at baseline. Unit costs were obtained from appropriate national published sources including the Information Services Division of the Scottish Government Scotland tariffs for 2012^2^, the Personal Social Science Research Unit^3^, the British National Formulary^3^ and, where necessary, NHS reference costs for 2011/12.

The CARE Plus consultations have been costed according to the length of the consultation with either the GP or practice nurse. Where details regarding the length of the CARE Plus consultations are missing the average length of consultations of the same type (first, second, third etc) was used. Other non-CARE Plus consultations were costed for an average 11.7 minutes (GP) or 15.5 minutes (Practice Nurse). Consultations where the patient did not attend (DNA) were costed at the same rate as for other consultations. Any over-the counter medications were excluded from the analysis due to the NHS and Personal Social Service perspective.

**Table 2. Intervention Costs – GP and Practice Nurse Training**

| **Type of cost** | **Cost** | **Unit** | **No.** | **Delivery** | **Participants** | **Total cost** | **Per patient** | **Source** |
| --- | --- | --- | --- | --- | --- | --- | --- | --- |
|  |  |  |  |  |  |  |  |  |
| GP | £129.00 | Hour | 3 | 3 | 9 | £ 10,449.00 | £ 137.49 | PSSRU 2012 |
| Practice nurse | £41.00 | Hour | 3 | 3 | 2 | £ 738.00 | £ 9.71 | PSSRU 2012 |
| Trainers | £129.00 | Hour | 3 | 3 | 2 | £ 2,322.00 | £ 30.55 | PSSRU 2012 |
| Materials | £20.00 | Pack | 1 | 1 | 11 | £ 220.00 | £ 2.89 | Trial |
| Venue hire | £30.00 | Session | 1 | 3 |  | £ 90.00 | £ 1.18 | Trial |
| Refreshments | £10.00 | Person | 1 | 3 | 13 | £ 390.00 | £ 5.13 | Trial |
| Self-help pack | £15.00 | Patient | 1 | 1 | 76 | £ 1,140.00 | £ 15.00 | Trial |
|  |  |  |  |  |  |  |  |  |
| **Roll out costs** |  |  |  |  |  | £ 15,349.00 | £ 201.96 |  |

***Outcome Measurement***

Self report EQ-5D-5L data were collected at baseline, 6 months and 12 months. These were converted into preference-based health utilities using the EQ-5D algorithm. The area under the curve (AUC) method was used to determine the overall utility over the trial period^5^. This provides a utility weighting, similar to a QALY, but measured over a 12 month period. The utility change over the year was calculated using all available data and using linear model regression methods that adjusted for the clustering of patients within practices as a random effect as well as age, sex and those baseline characteristics found to be significantly different between the groups. .

***Analysis***

The within-trial effectiveness is presented in terms of the QALYs gained over 12 months. Differences in the average utility change between the intervention and control groups give an estimate of the QALYs gained from the intervention, assuming no differences beyond the 12 month follow-up period. Effect results are presented as mean values with confidence intervals. The cost associated with each individual is determined as the sum of the intervention costs (for the CARE Plus intervention group) and the cost of NHS resource use. The change in costs over the year for each participant is then analysed in a generalised linear model (Gaussian with identity link) and adjusting for clustering and other covariates as described above . This ensures that any imbalance between the two groups at baseline is not reflected in the estimate of costs. The incremental change in cost associated with the CARE Plus intervention is the adjusted difference between the CARE Plus intervention group and the control group controlling for baseline costs. Cost results are presented as mean values. The incremental cost-effectiveness associated with the CARE Plus intervention is then presented in terms of incremental cost per QALY gained. Discounting of costs and outcomes is not required due to the 12 month time frame of the within trial analysis.

The uncertainty surrounding the estimates of incremental costs, incremental effects and cost-effectiveness was investigated through nesting the regressions adjusting for clustering and other covariates within a non-parametric bootstrap with 1,000 iterations undertaken in STATA v12. All other analysis and modelling was carried out in MS Excel. The uncertainty surrounding the cost-effectiveness results are presented on the cost-effectiveness plane and summarised on a cost-effectiveness acceptability curve.

**Results**

The total costs associated with the CARE Plus intervention group were estimated to be £312,449 compared to total costs for usual care of £243,793. The CARE Plus intervention group was associated with an increase in costs of £82,989), compared to a slight increase in costs for usual care of £487. As such the incremental cost of the CARE Plus intervention was £82,501 with an adjusted mean difference of £929, (95% CIs: £86, £1788) per participant.

| **TABLE 3** | **Intervention** | | | **Controls** | | |
| --- | --- | --- | --- | --- | --- | --- |
| **Cost categories** | **Baseline** | **12mths** | **Change in costs** | **Baseline** | **12mths** | **Change in costs** |
| Practitioner primary care consultations (1) | £50,013 | £45,489 | -£4,524 | £31,438 | £31,971 | £533 |
| Other healthcare services (2) | £5,817 | £7,370 | £1,553 | £12,578 | £12,698 | £120 |
| Outpatient | £36,379 | £34,505 | -£1,873 | £24,094 | £28,814 | £4,720 |
| Inpatient | £32,327 | £82,067 | £49,740 | £66,339 | £64,050 | -£2,289 |
| DNA practice consultations | £2,326 | £2,257 | -£69 | £2,852 | £3,360 | £508 |
| Lab and radiology tests | £4,455 | £3,722 | -£732 | £6,135 | £5,693 | -£442 |
| A&E | £2,600 | £4,368 | £1,768 | £3,640 | £3,536 | -£104 |
| Out of hours practioner | £2,433 | £3,372 | £939 | £2,977 | £2,314 | -£663 |
| Prescriptions (excluding OTC) | £93,110 | £99,383 | £6,272 | £93,253 | £91,357 | -£1,895 |
| Careplus consultations incl. GP training, materials etc | £0 | £29,916 | £29,916 | £0 | £0 | £0 |
| **Total** | **£229,460** | **£312,449** | **£82,989** | **£243,306** | **£243,793** | **£487** |
|  |  |  |  |  |  |  |
| (1) GP, Nurse, Other - in surgery, by telephone, home visits | |  |  |  |  |  |
| (2) Admin, data entry |  |  |  |  |  |  |

Table 4 presents the average change in costs and utility (per participant) in each group.

Table 4: Cost, effect and cost-effectiveness results (within trial analysis)

|  | Cost (£) | Utility change over 12 months |
| --- | --- | --- |
|  | | |
| Incremental | £ 929, standard error £452 | 0·076 (95% CI: 0·028, 0·124), standard error 0.026 |

The results illustrate that CARE Plus was more expensive than usual care with an additional cost of £929 per individual (95% CIs: £86, £1788)). This was driven partly by the additional cost of the intervention but more by a large increase in inpatient costs in the intervention group (increase of £654 per participant in the CARE Plus intervention group compared to a reduction of £30 per participant in the usual care group). The results also indicate that CARE Plus was more effective in terms of QALYs with a gain in quality adjusted life years of 0·076 (95% CI: 0·028, 0·124). As a result the CARE Plus intervention was associated with an incremental cost-effectiveness of £ 12,224 per QALY gained.

Figure 1 shows the uncertainty surrounding the estimates of the costs and effects on the incremental cost-effectiveness plane. The figure shows that there is very little uncertainty about whether CARE Plus was more expensive than usual care (i.e. almost all of the incremental costs are positive), but there is considerable uncertainty about the magnitude of the cost difference. In addition, the figure shows that there is almost no uncertainty about whether CARE Plus was more effective than usual care (i.e. almost all of the incremental QALYs are positive) but there is considerable uncertainty about the magnitude of the effect difference.

**Figure 1: Incremental cost-effectiveness plane for CARE Plus intervention compared to usual care (within trial analysis)**

The cost-effectiveness acceptability curve (Figure 2) illustrates the probability that the CARE Plus intervention was cost-effective for any given value of the cost-effectiveness threshold. For a cost-effectiveness threshold of £20,000/QALY, the probability that CARE Plus was cost-effective, compared to usual care, is 0.79. This probability rises to 0.93 for a cost-effectiveness threshold of £30,000/QALY.

**Figure 2: Cost-effectiveness acceptability curve for CARE Plus intervention (within trial analysis)**

**Lifetime analysis**

The within-trial cost-effectiveness analysis used actual trial data to estimate the cost-effectiveness of the CARE Plus intervention, compared to usual care, over the 12 month trial period. The analysis involves the assumption that there are no differences in QALYs between the intervention and control groups beyond the 12 month trial follow-up period. However, it is expected that the impact of the CARE Plus intervention will extend beyond the trial period, with impacts on the quality of life and health service costs of patients in the longer term. The aim of the lifetime analysis was to estimate the longer term implications (in terms of cost and effect) of the CARE Plus intervention in order to establish the long term cost-effectiveness of the intervention compared with usual care. The analysis involved extrapolating the short term impacts identified within the trial period through the use of a model.

**Methods**

The intention was to estimate the impact of CARE Plus in terms of quality of life and health service costs across the remaining life expectancy of the patients recruited to the trial. The following describes, in turn: how life expectancy projections were made for patients to take account of multimorbidity; how the trial findings regarding quality of life and costs were extrapolated across remaining life expectancy to estimate long term costs, effects and cost effectiveness; and how the analysis incorporated uncertainty.

***Estimating life expectancy***

The initial stage of the long term analysis involved estimating the life expectancy for the CARE Plus patient population. Life expectancy tables based on gender, age, SIMD quintile and no of conditions (defined as 2, 3 or 4+) were generated in a 3 part process, as follows:

First, general Scottish population lifetables defined by gender, 5-year age bands, and SIMD quintiles were identified from Scottish Public Health Observatory^6^. These tables refer to a 2003 population. As such, they were updated to a contemporary population by scaling-up projections to take account of the increase in life expectancy for the population as a whole from birth, using the latest estimates from the General Registry Office for Scotland.^7^

Second, the expected life years lost from living with 2, 3, or 4+ chronic conditions was estimated. Lin et al (2006) estimated the independent impact of a wide range of chronic conditions on life expectancy^8^. Under the assumption that as the number of conditions increases the cumulative impact on life years lost is additive, estimates of the reduction in life years lost from different combinations of 2, 3 and 4+ conditions were generated. This assumption is consistent with Lin et al’s approach to estimating the impact of individual conditions, which took into account the presence of other conditions. For simplicity, the different combinations of conditions were restricted to cancers, coronary heart disease, stroke, and respiratory disease. These conditions are the most prevalent in those suffering from multimorbidity in Scotland^9^ and are also the main causes of death in Scotland.^10^ Third, the estimates of life years lost were subtracted from the general Scottish population estimates of remaining life expectancy to produce separate tables of life expectancy based on gender, 5-year age bands and SIMD quintiles for those with 2, 3 and 4+ conditions. In order to avoid double counting the impact of multimorbidity, this analysis took account of the prevalence of multimorbidity in the general population. This was done via a set of weights representing the percentage in the general population suffering from multimorbidity by 5-year age group. These estimates came from Barnett et al (2012)^9^. Overall, this three stage process produced a set of tables that estimated the remaining life expectancy based on gender, 5-year age bands and SIMD quintiles for those with 2, 3 and 4+ conditions***.***

***Lifetime effect, lifetime cost and incremental cost effectiveness***

The second stage of the long term analysis involved estimating how the trial results observed at 12-months might be sustained across the remaining life expectancy of patients.

***Lifetime effect***

The within-trial analysis provided estimates of the average change in the health utility score over the 12 months for both the CARE Plus intervention and the usual care groups. As noted above, the difference in the average change in health utility score between the CARE Plus intervention group and the usual care group was 0·076 (95% CI: 0·028, 0·124) Three scenarios were generated to represent the possible long terms impacts of the CARE Plus intervention in terms of sustaining this impact on health utility over the patients remaining life expectancy. The ‘continuous impact’ scenario assumed that the improvement in health utility observed for the CARE Plus intervention group was sustained over 2 years. The ‘declining impact’ scenario assumed that the impact of the CARE Plus intervention on the health utility declined over the 2 years. The rate of this reduction was based on the decrease in health utility observed in the trial data, with the annual percentage reduction twice the level observed in the data between months 6 and 12. The ‘average impact’ scenario was a simple average between the other two scenarios, i.e. essentially a declining impact scenario with a lower rate of decline.

***Lifetime costs***

The impact of the CARE Plus intervention on lifetime costs was modelled using a similar approach to that employed to model the lifetime effect. The within-trial analysis provided estimates of the average change in the health care costs over the 12 months for both the CARE Plus intervention and the usual care groups. As noted above, the difference in the average change in costs between the CARE Plus intervention and usual care group at 12 months was observed to be £929, (95% CIs: £86, £1788) Three scenarios were generated to represent the possible long terms impacts of the CARE Plus intervention in terms of sustaining this impact on health care costs over the patients remaining life expectancy. The ‘continuous impact’ scenario assumed that the increase in health care costs observed for the CARE Plus intervention group was sustained over 2 years. The ‘declining impact’ scenario assumed that the impact of the CARE Plus intervention on the health care costs declined over these 2 years, at the same rate as was used for the decline in health utility impact. The ‘average impact’ scenario was a simple average between the other two scenarios, i.e. essentially a declining impact scenario with a lower rate of decline.

***Discounting***

The longer term estimates of life expectancy, QALYs and costs were all discounted at a rate of 3.5% in accordance with practice by the Scottish Medicine Consortium (SMC), following guidance by the National Institute for Clinical Excellence.^1^

# Analysis

The long term effectiveness of the CARE Plus intervention is presented in terms of incremental QALYs gained compared to usual care. These estimates are generated for each individual in the CARE Plus intervention group, based on their life expectancy, and include the average utility change experienced in the within trial period. The long term cost of the CARE Plus intervention is presented in terms of the incremental costs compared to usual care. These estimates are generated for each individual in the CARE Plus intervention group, based on their life expectancy, and include the average change in costs experienced in the within trial period. The incremental cost and effects of the CARE Plus intervention are calculated by averaging the change in costs and effects of the individual participants who received the CARE Plus intervention. The incremental cost-effectiveness associated with the CARE Plus intervention is then presented in terms of incremental cost per QALY gained.

The analysis was undertaken for all three scenarios (continuous, declining, and average) as described above.

# *Probabilistic analysis*

In order to estimate the uncertainty in the model estimates, we undertook a probabilistic sensitivity analysis allowing for uncertainty in the estimation of the change in the health utility and the change in the health care costs within the model. Running this analysis required simulating 1000 draws from the probability distributions for these parameters and using each of these 1000 draws to estimate the life expectancy, QALYs and lifetime costs for each of the participants in the CARE Plus intervention group, generating an estimate of the average incremental costs and incremental QALYs for the group for each draw.

The resulting uncertainty in the incremental costs and effects associated with the CARE Plus intervention is plotted on the incremental cost-effectiveness plane. The cost-effectiveness acceptability curve presents the uncertainty surrounding the cost-effectiveness of the CARE Plus intervention compared to usual care.

***Value of information analysis***

All decisions are made in the context of uncertainty about the “true” consequences of the decision. Any decision made on the basis of cost-effectiveness may turn out to be wrong i.e. the intervention is not actually cost-effective. Where this is the case resources used to provide the intervention are being wasted and QALYs are being lost. Research is valuable because it generates information and reduces uncertainty surrounding the decision and thus reduces the cost of the uncertainty. Value of information (VOI) analysis involves a formal assessment of the costs and consequences associated with the uncertainty surrounding a decision in order to determine the potential value associated with undertaking research that would reduce uncertainty and inform the decision in the future. The value of information is assessed by the difference in the value of a decision taken with the current level of information/uncertainty and the value of a decision taken with more information (less uncertainty). Where the cost of undertaking the research to collect the information is less than the value associated with the decision made with the improved information then the research is worthwhile. The expected value of perfect information (EVPI) is a specific type of analysis that estimates the value associated with eliminating all uncertainty surrounding a decision. As such it provides a maximum value for undertaking further research (as perfect information is never achieved) and provides a necessary condition to judge whether further research is potentially worthwhile. If the cost of further research is greater than the EVPI then further research can not be worthwhile. However, if the cost of further research is less than the EVPI then the research is *potentially* worthwhile. Determining whether specific research is worthwhile requires a comparison of the value of the actual reduction in uncertainty achieved by the specific research, this can be assessed through the expected value of sample information (EVSI), with the actual costs of the specific research.

Here, the uncertainty surrounding the decision to implement the CARE Plus intervention was formally assessed using the expected value of perfect information (EVPI) to determine the potential worth of undertaking further research. If the EVPI for CARE Plus exceeds the expected costs of additional research then it is potentially cost-effective to obtain more information by undertaking further research. An assessment of the worth of specific research was beyond the scope of this project.

**Results**

**Remaining life expectancy for patients with multimorbidity**

Table X and Y provides the estimates of remaining life expectancy for men and women, by 5-year age band, SIMD quintiles and by 2, 3 and 4+ conditions. Gradients are evident in the remaining life expectancy by gender, age, and SIMD quintiles (socioeconomic deprivation). That is, remaining life expectancy is lower for men than women, falls as age increases, and is lower in more socioeconomic deprived groups.

**Lifetime effect, lifetime cost and incremental cost effectiveness**

Table 5 presents the results of the probabilistic analysis for the 3 scenarios in terms of the average incremental lifetime costs and incremental QALYs per participant in each group.

| CAREPlus | Incremental Cost (£) | Incremental QALYs |
| --- | --- | --- |
| Continuous | £1651 | 0.150 |
| Declining | £1147 | 0.098 |
| Average | £1399 | 0.124 |

Table 5: Average incremental lifetime costs and QALYs

The results illustrate that CARE Plus is more expensive than usual care. The results also indicate that the CARE Plus intervention is more effective than usual care. As a result the CARE Plus intervention is associated with an incremental cost-effectiveness of £11,319 per QALY gained.

Figure 3 a-c shows the uncertainty surrounding the estimates of the lifetime incremental costs and effects for the 3 scenarios. The figures show that for all of the scenarios there is very little or no uncertainty surrounding the existence of a cost difference: CARE Plus is generally more expensive than usual care although there are some negative incremental costs. There is however, considerable uncertainty about the magnitude of the cost difference. In addition, the figure shows that there is no uncertainty surrounding the existence of a difference in effect (i.e. all the incremental QALYs are positive), although there is uncertainty about the magnitude of the difference.

Figure 3a: Incremental Cost-Effectiveness Plane – continuous scenario

Figure 3b: Incremental Cost-Effectiveness Plane – declining scenario

Figure 3c: Incremental Cost-Effectiveness Plane – average scenario

Figure 4 presents the cost-effectiveness acceptability curve for the lifetime analysis. This illustrates the probability that the CARE Plus intervention is cost-effective for any given value of the cost-effectiveness threshold. For a cost-effectiveness threshold of £20,000/QALY, the probability that CARE Plus is cost-effective, compared to usual care, is 0.84. This probability rises to 0.96 for a cost-effectiveness threshold of £30,000/QALY.

Figure 4: Cost-effectiveness acceptability curve for CARE Plus intervention (lifetime analysis)

Figure 5 presents the expected value of perfect information associated with the decision between CARE Plus and usual care for each scenario. The values are presented for each instance of the decision (i.e. for every individual with multimorbidity who could be considered for CARE Plus instead of usual care). If the decision maker were willing to pay £20,000 per QALY, then perfect information would be worth between £73 per individual (declining scenario) and £94 per individual (continuous scenario). If the decision maker were willing to pay £30,000 per QALY, then perfect information would be worth between £16 per individual (declining scenario) and £20 per individual (continuous scenario).

Figure 5: Expected value of Perfect Information for each individual with multimorbidity

**Discussion**

The short term, within trial analysis demonstrated that the CARE Plus intervention was inexpensive to deliver, with an additional cost of just £929 per participant, with the increase in costs driven by increased inpatient costs associated with the intervention group. The short term, within trial analysis also indicated that CARE Plus is more effective than usual care in terms of QALYs (with a utility change of 0.076 over 12 months. As such, the within trial analysis demonstrated that the CARE Plus intervention was cost-effective, with an incremental cost of £12,224 per QALY gained. As noted above, a within-trial analysis assumes that there are no differences in costs and QALYs between the groups beyond the trial follow-up period. Here, a second analysis extrapolated the trial results to estimate the additional costs and benefits which might accrue over the participants’ lifetimes in order to provide an estimate of the lifetime cost-effectiveness of CARE Plus compared with usual care. The model projects life expectancy, incremental utility adjusted life expectancy and incremental lifetime costs based on the age, gender, SIMD quintile and number of conditions (2,3, or 4+). The lifetime analysis demonstrates that CARE Plus remains more expensive than usual care with an average additional cost of between £1,147 per individual (declining scenario) and £1,651 per individual (continuous scenario). However, the results also indicate that the CARE Plus intervention remains more effective with an average increase of between 0.098 QALYs (declining scenario) and 0.150 (continuous scenario). As a result the CARE Plus intervention is associated with an incremental cost-effectiveness of £ 11,319 per QALY gained. This is well below the threshold range of £20,000-£30,000 per QALY used by NICE^1^ when deciding whether an intervention provides good value for money to the NHS. The cost-effectiveness planes illustrate that there is no uncertainty, associated with any of the scenarios, that the CARE Plus intervention is more costly and more effective than usual care, however they all show that there is considerable uncertainty around the magnitude of the cost and effect increases. This uncertainty in the incremental cost and incremental effects associated with the CARE Plus intervention, compared to usual care, translates into uncertainty in the cost-effectiveness of CARE Plus, as illustrated by the cost-effectiveness acceptability curve. For a cost-effectiveness threshold of £20,000/QALY, the probability that CARE Plus is cost-effective, compared to usual care, is 0.84. This probability rises to 0.96 for a cost-effectiveness threshold of £30,000/QALY. This uncertainty translates into an expected value of perfect information , the absolute maximum amount that further research would be worth, of between £73-£94 per individual at a cost-effectiveness threshold of £20,000/QALY. This value drops to between £16-20 per individual for a cost-effectiveness threshold of £30,000/QALY, reflecting the reduction in the uncertainty at this threshold. These per individual amounts could be scaled up according to the frequency of the decision, in order to provide an estimate of the maximum return achievable, for the population, from reducing uncertainty through further research. This value would then be compared to the cost of undertaking further research to determine whether it is potentially worthwhile undertaking further research around the effectiveness or cost-effectiveness of CARE Plus compared to usual care.

The analyses have a number of strengths and limitations. Where possible the analysis utilises data from the Scottish population, for example in the estimation of the projections of life expectancy, as the source of cost inputs and to reflect the proportion living with multimorbidity. The projection of lifetime costs and QALYs is limited to an estimation of the additional costs and QALYs gained by the CARE Plus group compared to the usual care group based on the differences observed in the trial at 12 months. Uncertainty in the long term projections of additional costs and effects is represented in terms of the estimation of 3 scenarios (continuous, declining and average) which differ in terms of the possible long terms impacts of the CARE Plus intervention in terms of sustaining change in health utilities and health care costs. In addition, a probabilistic sensitivity analysis and value of information analysis estimate the impact of uncertainty in the levels of the change in health utility and health care costs. The CARE Plus trial was relatively small with 152 patients in total (76 in the CARE Plus intervention and usual care groups respectively) and there is considerable heterogeneity within the patient group in terms of age, SIMD and no of conditions. Given the sample size, sub-group analysis was not undertaken but future studies may provide data that would enable analysis of which patient sub-groups benefit most from CARE Plus.

The analysis presented here is among the first to estimate the long term cost-effectiveness of interventions in patients with multimorbidity. A review by Richardson et al (2005) of interventions to support self care found that that there is no robust and consistent evidence to support any general conclusion^12^. Studies were limited in that the study design was typically poor and the majority of the 13 studies that claimed to present an economic evaluation were actually cost consequence analyses. In an update to this review, we found five more studies, all randomised clinical trials, investigating the cost effectiveness of self management interventions (see Table Z). All of the studies used QALYs, derived using the EQ-5D-3L. Two of these studies focussed on patients with multimorbidity: the Expert Patient Programme^13^ (Richardson, 2008) and lay-led self support groups^14^ (Kennedy, 2007). Both were shown to be cost effective. None of the five studies examines the long term impact of the interventions, with all of the studies restricted to within-trial evaluations of 6 or 12 month duration.

Taking all these considerations into account, our analyses suggest that the CARE Plus intervention provides value for money in both the short and longer term and that there would potentially be value in undertaking further research in the form of a larger RCT investigating the longer term impact of CARE Plus on different patient sub-groups.

**Table X Remaining life expectancy – males**

**Table Y Remaining life expectancy – females**

| Year/Author | Conditions | Population | Intervention | Study Design/Follow up | Outcome measure(s) | Cost-effectiveness Findings |
| --- | --- | --- | --- | --- | --- | --- |
| Richardson (2006)^15^ | Inflammatory Bowel Disease | England | Whole systems self-management | RCT/ 1 year | QALYs  (EQ-5D-3L used) | +ve  Probability cost effective of 61%  (reduced costs/ increased QALY) |
| Hurley (2007)^16^ | Chronic knee pain | England | Rehab and self management  Comparison of Individual vs group rehab | RCT/ 6 months | QALYs  (EQ-5D-3L used) | +ve  Probability cost effective: 90%  Individual rehab more cost effective as WTP increased |
| Kennedy (2007)^14^ | Multiple | England | Lay-led self support groups | RCT / 6 months | QALYs  (EQ-5D-3L used) | +ve  Probability of 70% |
| Richardson (2008)^13^ | Multiple | England | Expert patients  programme | RCT/ 6 months | QALYs  (EQ-5D-3L used) | + ve  Probability of 94% |
| Patel (2009)^17^ | Arthritis | UK | Self management and education booklet | RCT/ 1 year | QALYs  (EQ-5D-3L used) | -ve  Low probability of being cost effective |

* Probability of cost effectiveness varies as the willingness to pay an extra QALY changes. Reported probabilities in the table are conditional upon NICE’s minimum threshold of £20k per QALY.

**Table Z: Review of self-care cost effectiveness studies (post-2005)**

**References**

1. Guide to the methods of technology appraisal 2013: NICE National Institute for Health and Care Excellence; 04 April 2013.
2. Information Services Division Scotland. <http://www.isdscotland.org/>. Accessed 16th August, 2013.
3. Personal Social Services Research Unit. Unit Cost of Health and Social Care. 2011; <http://www.pssru.ac.uk/project-pages/unit-costs/2011/index.php>. Accessed 16th August, 2013.
4. British National Formulary. [http://www.bnf.org/bnf/index.htm. Accessed 16 August 2013](http://www.bnf.org/bnf/index.htm.%20Accessed%2016%20August%202013).
5. Manca A, Hawkins N, Sculpher MJ. Estimating mean QALYs in trial-based cost-effectiveness analysis: the importance of controlling for baseline utility. *Health Econ.* 2005;14(5):487-496
6. Scottish Public Health Observatory, Healthy Life Expectancy. <http://www.scotpho.org.uk/population-dynamics/healthy-life-expectancy/data/deprivation-quintiles> (accessed December 2013)
7. General Registrar Office for Scotland (GROS) (2013), Interim life tables 2011 <http://www.gro-scotland.gov.uk/statistics/theme/life-expectancy/scotland/interim-life-tables.html> (accessed December 2013)
8. Lin et al, Decomposition of life expectancy and expected life-years lost by disease, Statistics in Medicine, 2006; 25:1922–1936
9. Barnett et al, Epidemiology of multimorbidity and implications for health care, research, and medical education: a cross-sectional study. The Lancet, [Volume 380, Issue 9851](http://www.thelancet.com/journals/lancet/issue/vol380no9851/PIIS0140-6736(12)X6043-4), Pages 1382 - 1383, 20 October 2012
10. General Registrar Office for Scotland (GROS) (2012), Vital events reference tables. <http://www.gro-scotland.gov.uk/statistics/theme/vital-events/general/ref-tables/2012/index.html> (accessed December 2013
11. **Richardson G et al** Cost-effectiveness of interventions to support self-care: a systematic review. International Journal of Technology Assessment in Health Care. 2005;21(4):423-32.
12. [Richardson](http://jech.bmj.com/search?author1=G+Richardson&sortspec=date&submit=Submit) et al Cost effectiveness of the Expert Patients Programme (EPP) for patients with chronic conditions[^1^](http://jech.bmj.com/content/62/4/361.short#aff-1)*J Epidemiol Community Health* 2008;62:361-367 doi:10.1136/jech.2006.057430
13. [Kennedy](http://jech.bmj.com/search?author1=Anne+Kennedy&sortspec=date&submit=Submit)[^1^](http://jech.bmj.com/content/61/3/254.abstract#aff-1) et al , The effectiveness and cost effectiveness of a national lay-led self care support programme for patients with long-term conditions: a pragmatic randomised controlled trial *J Epidemiol Community Health* 2007;61:254-261
14. **Richardson G,** Sculpher M, Kennedy A, Nelson E, Reeves D, Roberts C, et al. Is self-care a cost-effective use of resources? Evidence from a randomised trial in inflammatory bowel disease. Journal of Health Services Research and Policy 2006;11(4):225-30.
15. [Hurley MV](http://www.ncbi.nlm.nih.gov/pubmed?term=Hurley%20MV%5BAuthor%5D&cauthor=true&cauthor_uid=17907147) et al , Clinical effectiveness of a rehabilitation program integrating exercise, self-management, and active coping strategies for chronic knee pain: a cluster randomized trial. [Arthritis Rheum.](http://www.ncbi.nlm.nih.gov/pubmed/17907147) 2007 Oct 15;57(7):1211-9.
16. Patel A et al, Economic evaluation of arthritis self management in primary care, BMJ *2009;* *339*
